# Safety aspects of hysteroscopy, specifically in relation to entry and specimen retrieval: a UK survey of practice

**DOI:** 10.1186/s10397-018-1036-6

**Published:** 2018-01-15

**Authors:** S. H. Walker, L. Gokhale

**Affiliations:** Department of Obstetrics and Gynaecology, Royal Gwent Hospital, Aneurin Bevan University Health Board, Newport, UK

**Keywords:** Hysteroscopy, Uterine perforation, Specimen retrieval

## Abstract

**Background:**

The purpose of this study is to evaluate current practice amongst gynaecologists across the UK, regarding safety aspects of inpatient hysteroscopy under anaesthesia, specifically in relation to entry and specimen retrieval.

A survey was created using survey monkey. The first round was circulated to all registrar trainees and consultant gynaecologists across Wales. Following a good response, the survey was then circulated to all members of the British Society of Gynaecological Endoscopy (BSGE).

**Results:**

There were 212 responses including, 140 consultants, 36 senior registrars, 17 junior registrars and 18 clinical nurse specialists. In total, 136 out of 212 (64.7%) always perform a vaginal examination prior to hysteroscopy. 10.4% always sound the uterus, and 5.2% always dilate the uterus prior to insertion of the hysteroscope. Twenty-three consultants, six senior registrars, three junior registrars and one clinical nurse specialist knew how to position the internal cervical os as visualised through the scope when using a 30° hysteroscope. 35.8% of candidates always perform a post-procedure cavity check, and 9% use suction to flush the cavity to aid vision during the post-procedure cavity check. The majority (76%) predicted dilatation as the stage most likely to cause uterine perforation and predicted the most likely site for perforation as the posterior uterine wall in the anteverted uterus and the anterior uterine wall in the retroverted uterus.

**Conclusion:**

This study highlights varied practice across the UK regarding safety aspects of hysteroscopy, in relation to entry and specimen retrieval. There is a need for increased awareness of the risks of hysteroscopy and paramount precautions that should be performed routinely as part of their practice. Standardised guidelines may be a beneficial tool to help bring about this change in practice, leading to a reduction in uterine perforation rates.

## Background

The hysteroscope has become a standard part of a gynaecologists’ armamentarium, with operative hysteroscopy increasing as a surgical alternative for various gynaecological problems [1]. Uterine perforation is an uncommon but potentially serious complication of hysteroscopy. Guidance from the Royal College of Obstetricians and Gynaecologists (RCOG) on best practice in outpatient hysteroscopy estimates a perforation rate of 0.007–1.7% [2]. With higher rates of 1.6% reported for operative hysteroscopy [3, 4]. Risk factors include cervical stenosis, tortuous cervical canal and deviated uterine cavity as a result of fibroids [2, 5].

Outpatient hysteroscopy with or without the use of local anaesthesia is now an established technique [1]. It is associated with a lower incidence of uterine trauma due to being performed with smaller-diameter hysteroscopes and under direct vision [2]. The main reasons for failure to successfully perform outpatient hysteroscopy includes cervical stenosis, severe pain, vasovagal reaction and high body mass index (BMI) making access difficult [2, 6]. Consequently, there will always be a necessity for inpatient hysteroscopy under general anaesthetic, and this cohort of women are at higher risk of complications due to increasing operative complexity, higher incidence of cervical stenosis, postmenopausal cervical atrophy and co-morbidities [7].

It has been estimated that 55% of uterine perforations are entry related (i.e. secondary to sounding, dilatation and insertion of hysteroscope) and 45% are related to technique used and improper use of the probe [1, 8].

Hysteroscopy carries small risks that cannot be eliminated completely, but preventing hysteroscopy complications starts by raising awareness of risks and precautions [1]. Currently there are no clear guidelines regarding safety aspects of inpatient diagnostic and operative hysteroscopy under general anaesthetic.

The aim of this study is to evaluate current practice amongst gynaecologists across the UK regarding safety aspects of inpatient hysteroscopy under general anaesthetic, specifically in relation to entry and specimen retrieval. Results from the survey may help determine if practice needs to change and whether there is a need for standardised guidelines on inpatient hysteroscopy under general anaesthetic.

## Method

A survey was created using survey monkey. The first round was circulated to all gynaecological speciality-training registrar doctors in their third to seventh year of training (ST3-7 trainees) and consultant gynaecologists across Wales in December 2016. Following a good response to this, the survey was then circulated to all members of the British Society of Gynaecological Endoscopy (BSGE) in June 2017. No ethical approval was required as the survey was optional and anonymous and study aims explained to all candidates prior to performing the survey.

Questions were based on safety aspects of all the stages of hysteroscopy, which can lead to uterine perforation. The survey specified for inpatient hysteroscopy under general anaesthetic. Question 1 was whether they routinely perform a vaginal examination prior to hysteroscopy. The next four questions were entry-related; whether they routinely sound and dilate the uterus prior to entry of the hysteroscope, the type of hysteroscope used and technique used when inserting the hysteroscope. Question 6 related to specimen retrieval. Questions 7 and 8 were whether they perform a post-procedure cavity check and whether they use suction to flush the cavity to aid this step.

An additional three questions were included in the survey when it was circulated to members of the BSGE. These included; the anatomical location the candidate thought you are most likely to perforate during hysteroscopy, during which stage of hysteroscopy they are most likely to perforate, and finally whether they use a standard proforma for documentation of their findings in their department.

Results were collated on an excel spreadsheet and analysed. For analysis the grades were split up into consultants, senior registrars (ST5-7), junior registrars (ST3-4) and clinical nurse specialists.

## Results

In total, 212 responses were included in analysis, 83 out of 170 responses (48.8%) from the first round to gynaecologists across Wales (13 junior registrars, 15 senior registrars, 55 consultants) and 129 out of 983 responses (13.1%) from the second round to members of the BSGE covering all regions of the UK (18 clinical nurse specialists, 5 junior registrars, 21 senior registrars, 85 consultants).

### Hysteroscopic approach and entry-related safety precautions

As shown in Table 1, in total, 64.2% (136/212) always carry out a vaginal examination prior to hysteroscopy, with a higher proportion of junior registrars (88.9%) compared to consultants (59.3%) always carrying out vaginal examination prior to hysteroscopy. In total, 10.4% (22/212) always sound the uterus before inserting the hysteroscope (15 consultants, 2 senior registrars, 4 junior registrars and 1 clinical nurse specialist). In total, 5.2% (11/212) always dilate, 22.6% (48/212) never dilate and 72.2% (153/212) sometimes dilate before inserting the hysteroscope (Table 1).

**Table 1:** Summary of response to questions related to safety aspects of hysteroscopy

Questions	Response	Number (percentage)				
		Consultants	Senior registrar	Junior registrar	Clinical nurse specialist	Total
Do you carry out a vaginal examination before hysteroscopy?	Always	83 (59.3)	31 (86.1)	16 (88.9)	6 (33.3)	136 (64.2)
	Never	7 (5)	1 (2.8)	0	2 (11.1)	10 (4.7)
	Sometimes	50 (35.7)	4 (11.1)	2 (11.1)	10 (55.6)	66 (31.1)
Do you Sound the uterus before inserting the hysteroscope?	Always	15 (10.7)	2 (5.6)	4 (22.2)	1 (5.6)	22 (10.4)
	Never	77 (55)	20 (55.6)	8 (44.4)	13 (72.2)	118 (55.7)
	Sometimes	48 (34.3)	14 (38.9)	6 (33.3)	4 (22.2)	72 (34)
Do you dilate before inserting the hysteroscope?	Always	7 (5)	3 (8.3)	1 (5.6)	0	11 (5.2)
	Never	36 (25.7)	8 (22.2)	1 (5.6)	3 (16.7)	48 (22.6)
	Sometimes	97 (69.3)	25 (69.4)	16 (88.9)	15 (83.3)	153 (72.2)
Following collection of specimen, do you carry out post-procedure cavity check?	Always	63 (45)	7 (19.4)	4 (22.2)	2 (11.1)	76 (35.8)
	Never	12 (8.6)	4 (11.1)	2 (11.1)	9 (50)	27 (12.7)
	Sometimes	65 (46.4)	25 (69.4)	12 (66.7)	7 (38.9)	109 (51.4)
When carrying out post-procedure cavity check, do you use suction to flush the cavity?	Always	16 (11.4)	2 (5.6)	1 (5.6)	0	19 (9)
	Never	84 (60)	22 (61.1)	13 (72.2)	17 (94.4)	136 (64.2)
	Sometimes	40 (28.6)	12 (33.3)	4 (22.2)	1 (5.)	57 (26.9)

*N* = 212, with percentages in brackets. Grades of candidates are divided up in the columns

When asking which type of hysteroscope candidate’s use, the majority of registrars never use a 0° hysteroscope, with 13.6% of consultants and 33.3% of clinical nurse specialists only using a 0° hysteroscope. Table 2 summarises the responses given when candidates were asked how they position the internal cervical os as visualised through the scope when using a 30° scope. Only 16.7% (34/204) knew the correct position being, ‘the 6 o’clock position for anteverted uterus and 12 o’clock position for retroverted uterus.’ The commonest answer given by 34.8% (71/204) was ‘always the 6 o’clock position’, which is the correct answer for an anteverted uterus, followed by ‘the way the hysteroscope naturally goes’, for which 32.4% gave as their answer (66/204).

**Table 2:** Summary of response by candidates to the question of how they position the internal cervical os as visualised through the scope during insertion of a 30° hyster scope

How do you position the internal cervical os as visualised through the 30° hysteroscope?	Number (percentage)				
	Consultants	Senior registrar	Junior registrar	Clinical nurse Specialist	Total
Always 6 o’clock position	42 (30.2)	15 (44.1)	8 (44.4)	6 (46.2)	71 (34.8)
Always 12 o’clock position	12 (8.6)	4 (11.8)	0	1 (7.7)	17 (8.3)
The way the hysteroscope naturally goes	52 (37.4)	6 (17.6)	3 (16.7)	5 (38.5)	66 (32.4)
6 o’clock position for anteverted uterus, 12 o’clock position for retroverted uterus	23 (16.5)	6 (17.6)	4 (22.2)	1 (7.7)	34 (16.7)
12 o’clock position for anteverted uterus, 6 o’clock position for retroverted uterus	10 (7.2)	3 (8.8)	3 (16.7)	0	16 (7.8)

*N* = 204, with the percentages in brackets. Grades of candidates are divided up in the columns

### Hysteroscopic technique

Table 3 demonstrates the instruments used by candidates for specimen retrieval. The majority of candidates use a range of instruments depending on availability. Overall, more candidates used polyp forceps (71%) and curette (58%), both being blind procedures compared to specimen retrieval under direct vision including myosure (24%) and resectoscope (10%).

**Table 3:** Summary of instruments used for specimen retrieval during hysteroscopy

Instrument used for specimen retrieval	Consultant	Senior registrar	Junior registrar	Clinical nurse specialist	Total
Polyp forceps	49	9	6	7	71
Currette	38	9	6	5	58
Versapoint	15	1	0	0	16
Myosure	19	1	0	4	24
Pipelle	8	0	0	5	13
All of above 5 options depending on availability	61	25	9	3	98
Resectoscope	7	2	0	1	10
Truclear	4	0	0	0	4

Candidates were able to give one or more responses and the columns separate out the different grades of gynaecologists

As shown at the bottom of Table 1, in total, 35.8% candidates (76/112) always carry out post-procedure cavity checks. A higher proportion of consultants (45%) carry out post-procedure cavity checks compared to junior registrars (22.2%) and clinical nurse specialists (11.1%). Only 9% of candidates (19/212) use suction to flush the cavity to aid vision during the post-procedure cavity checks (16 consultants, 2 senior registrars and 1 junior registrar).

**Fig. 1:**
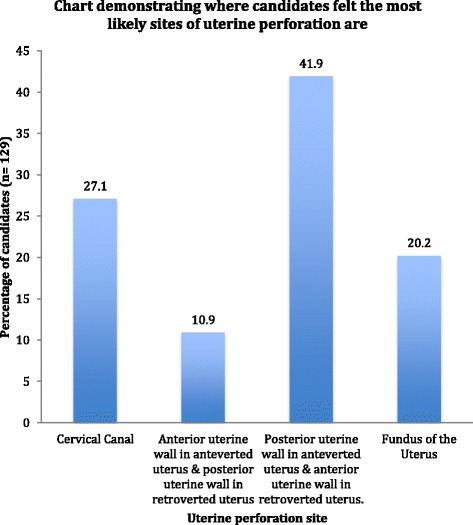
Chart demonstrating where candidates (in percentages) felt the most likely anatomical location of uterine perforation is

**Fig. 2:**
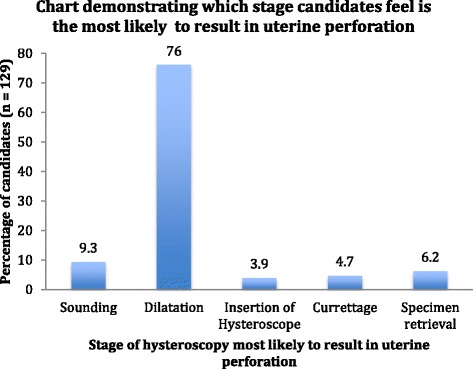
Chart demonstrating the percentage of candidates who predicted which stage of hysteroscopy is the cause behind most uterine perforations seen

For the additional three questions asked to the 129 candidates from the BSGE, the biggest response given by 41.9%; when asked what anatomical location they felt you are most likely to perforate during hysteroscopy, was the ‘posterior uterine wall in the anteverted uterus and the anterior uterine wall in the retroverted uterus’ (Fig. 1). The majority (76%) predicted dilatation as the stage of hysteroscopy most likely to cause uterine perforation (Fig. 2). Lastly, 41.1% (53/129) use a standard proforma for documentation of their findings following hysteroscopy.

## Discussion

### Main findings

The results from this survey show varied practice amongst gynaecologists across the UK. This is reflected through limited information being available in the literature regarding safety aspects of hysteroscopy, specifically in relation to entry and specimen retrieval. There are guidelines available from the RCOG on best practice in outpatient hysteroscopy [2], but no guidelines available on inpatient diagnostic and operative hysteroscopy under general anaesthetic.

When asked if candidates perform a vaginal examination prior to hysteroscopy, 64.2% stated that they always do, 31.1% sometimes and 4.7% never. Literature highlights bimanual assessment being a vital step to perform to correctly identify the size, position and attitude of the uterus, helping to determine the direction to insert the hysteroscope, and reducing the risk of uterine perforation [9–11]. However, if the patient has had an ultrasound scan indicating the uterine position then this step may be omitted, provided the surgeon is aware of the ultrasound report.

Whether or not to sound the uterus prior to hysteroscopy is debatable, with limited evidence for its use in literature. In the survey, of concern, 10.4% of candidates stated that they always sound the uterus prior to insertion of the hysteroscope. Some articles report it as a useful step to help determine the length and direction of the internal os and uterine cavity, thereby reducing your chance of perforation and suspecting perforation when the sound goes beyond the expected size of the uterus [10–12]. However, it is another instrument introduced into the uterus, increasing the risk of perforation and should only be used on occasion with proper technique of a gentle approach holding the sound like a pen, not a skewer to avoid perforation [10].

Dilatation is reported to be when most cervical trauma and uterine perforations occur [5]. One study reported 50% of their perforations occurred during dilatation of the cervix [8]. Dilatation is not recommended for diagnostic procedures [1]. However, gradual cervical dilatation is sometimes required in cervical stenosis. Articles report the importance of avoiding excessive force and the use of half-size dilators to reduce the risk of perforation [9]. In the present study, candidates were aware of the risk of dilatation with 76% determining dilatation to be the commonest step to result in uterine perforation; however, 5.2% stated they always perform dilatation prior to insertion of the hysteroscope (Fig. 2).

**Fig. 3:**
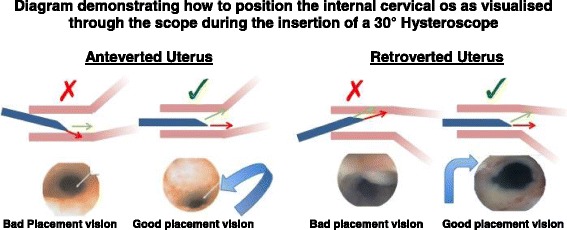
Diagram demonstrating how to position the internal cervical os as visualised through the hysteroscope during insertion of the 30° hysteroscope. The image to the left demonstrates the technique with an anteverted uterus to guide the hysteroscope along the posterior cervical wall keeping the internal os at the 6 o’clock position. The image to the right demonstrates the technique with a retroverted uterus to guide the hysteroscope along the anterior cervical wall keeping the internal os at the 12 o’clock position

There is limited information available on how to position the internal cervical os as visualised through the scope when inserting a 30° hysteroscope. This was reflected in the study with only 16.7% knowing the correct technique. The correct technique to avoid perforation, for an anteverted uterus, is to guide the hysteroscope along the posterior cervical wall keeping the internal os at the 6 o’clock position and in a retroverted uterus to guide the hysteroscope along the anterior cervical wall keeping the internal os at the 12 o’clock position (see Fig. 3). The correct technique not only allows for a smooth procedure but also prevents perforation and creation of a false passage [13]. A review article titled, ‘the perforated uterus’ published in 2013 highlights the commonest site of uterine perforation being the anterior uterine wall, which was also the commonest response given by candidates in this study [9]. This corresponds to the site of perforation in the retroverted uterus which being the less common uterine anatomy often catches the gynaecologist off guard, highlighting the importance of good technique in determining the attitude of the uterus to avoid this mistake.

Continual advancement in technology and improvement in surgical instruments has resulted in a range of instruments available for specimen retrieval. Some instruments including polyp forceps and curette have a higher chance of uterine perforation due to being a blind procedure compared to myosure and resectoscope, which are performed under direct vision. The survey demonstrates a range of instruments used resulting from different health board funds and training of the staff. It also reflects limited knowledge of the risks of using different instruments.

Post-procedure cavity checks aid identification of an unsuspected uterine perforation. Sudden loss of vision during hysteroscopic procedures due to collapse of the uterus and bleeding together with a large deficit of distension medium is highly suggestive of uterine perforation [9, 11]. In this survey, only 35.8% always perform a post-procedure cavity check and 9% always use suction to aid vision during a post-procedure cavity check.

The study shows a range in practice amongst the different groups of practitioners. In general, a higher proportion of junior and senior registrars (88.9 vs 86.1%) always perform a vaginal examination (VE) compared to the consultants (59.3%) and nurse specialists (33.3%). This is expected as consultants through years of experience, performing multiple hysteroscopies in their working day, do not necessarily perform a VE as they know what to expect. Double the proportion of junior registrars (22.2%) always sound the uterus compared to 10.7% consultants, which might reflect what is being taught to the juniors. A higher proportion of consultants compared to registrars and nurse specialists perform post-procedure cavity checks. This would be expected, as consultants are more likely to do complex operative hysteroscopy with higher risk of perforation.

### Strengths and limitations

This study provides a good overview about a national group of physicians and their methods of work. We were looking very specifically at entry techniques and specimen retrieval and hence the questionnaire focussed on this aspect. We did not include questions regarding other complications such as fluid absorption and its implications.

Limitations include a bias towards the practice in Wales as 39% of the responses were from Wales. Even though the study focused on inpatient hysteroscopy under general anaesthetic, it would have been good to expand on the differences in practice between outpatient hysteroscopy under local anaesthetic and inpatient hysteroscopy under general anaesthetic. Outpatient hysteroscopy uses a vaginoscopic approach; therefore, they are less likely to perform vaginal examination, sounding and dilatation and more likely to use ultrasound. This would help to also differentiate the difference between diagnostic and operative hysteroscopies.

## Conclusion

The study highlights varied practice across the UK regarding safety aspects of hysteroscopy, in relation to entry and specimen retrieval. Some gynaecologists are still using questionable techniques. The high percentage of gynaecologists who sound and/or dilate the cervix before hysteroscopy, and the low rate of specialists who correctly know how to position the internal cervical os on the hysteroscope was surprising and raises the question of whether the juniors are being taught the correct techniques. There is a need for increased awareness of the risks of hysteroscopy and paramount precautions that should be performed routinely as part of their practice. Standardised guidelines regarding safety aspects of inpatient diagnostic and operative hysteroscopy, taking into account patient caveats, may be a beneficial tool to help bring about this change in practice, leading to a reduction in uterine perforation rates.

## Abbreviations

BMI: Body mass index
BSGE: British Society of Gynaecological Endoscopy
RCOG: Royal College of Obstetricians and Gynaecologists
ST3-7: Speciality-training registrar doctors in their 3rd to 7th year of training
UK: United Kingdom
